# Bifunctionalized Allenes. Part XVI. Synthesis of 3-Phosphoryl-2,5-dihydrofurans by Coinage Metal-Catalyzed Cyclo-isomerization of Phosphorylated α-Hydroxyallenes

**DOI:** 10.3390/molecules20047263

**Published:** 2015-04-21

**Authors:** Valerij Ch. Christov, Ismail E. Ismailov, Ivaylo K. Ivanov

**Affiliations:** Department of Organic Chemistry & Technology, Faculty of Natural Sciences, Konstantin Preslavsky University of Shumen, 115, Universitetska str., BG-9712 Shumen, Bulgaria; E-Mails: ismail78@mail.bg (I.E.I.); iivanov@shu-bg.net (I.K.I.)

**Keywords:** phosphorylated α-hydroxyallenes, cycloisomerization, coinage metal catalysts, 2,5-dihydrofurans

## Abstract

Phosphorylated α-hydroxyallenes **1** and **2** were smoothly converted into the corresponding 2,5-dihydrofurans **3** and **4** in an 5-*endo-trig* cycloisomerization reaction by using 5 mol % of coinage metal salts as catalyst. Experimental conditions such as the type of the solvent, the reaction temperature, the mol % and the type of the catalyst were optimized. This mild and efficient cyclization method can be applied to dimethyl 1-hydroxyalkyl-alka-1,2-dienephosphonates **1** and 2-diphenylphosphinoyl-2,3-dien-1-ols **2a**–**c** and 3-diphenylphosphinoyl-3,4-dien-2-ols **2d**,**e**, furnishing 3-phosphorylated 2,5-dihydrofurans **3** and **4** in very good yields.

## 1. Introduction

2,5-Dihydrofurans and their derivatives are structural subunits frequently found in a wide variety of natural products which find application as flavor and fragrance compounds and pharmaceuticals [1,2,3] and represent pivotal structural elements in a wide variety of different biologically active molecules. For instance, they can be found in mycotoxins such as verrucosidine [4] and the structurally related citreoviridine [5], as well as vitamin A metabolites [6], polyether antibiotics [7,8], spiroketals [9] and even amino acids [10]. 2,5-Dihydrofurans are also important intermediates in organic synthesis due to the presence of the C=C bond as well as the five-membered ring. Consequently, much attention has been paid to the development of efficient and diverse synthetic methods for construction of this five-membered ring system [11,12,13].

Transition metal-catalyzed cyclization of functionalized allenes bearing a nucleophilic center has attracted considearble attention in recent years [14]. Particularly, the cyclization reactions of allenols catalyzed by Ag(I) [15,16,17,18], Hg(II) [19,20], Pd(0) [21,22,23], Pd(II) [24,25], or Ru(III) [26,27] have become quite useful methodologies for the synthesis of five-, or six-membered oxygen-containing heterocycles. Krause’s group has reported a highly efficient and stereoselective synthesis of 2,5-dihydrofurans by Au(I)- and Au(III)-catalyzed [28,29,30,31,32,33] cycloisomerization of α-hydroxyallenes [34,35,36]. Moreover, the method is not restricted to the cycloisomerization of α-hydroxyallenes to 2,5-dihydrofurans [34,35], rather, it was recently extended by Krause’s group to the corresponding endo-cyclization of β-hydroxyallenes [37], α-/β-aminoallenes [37,38,39], and α-thioallenes [40] to the corresponding five- or six-membered *O*-, *N*-, or *S*-heterocycles. The method of choice, however, is the use of transition metal catalysts since this combines high reactivities and excellent yields with a tolerance to many functional groups. 

On the other hand, the literature data on the reactions of phosphorylated allenes with electrophilic reagents reveal that the reactions proceed with cyclization of the allenic system bearing the phosphoryl group (O=P-C=C=C) to give heterocyclic compounds in most cases and the outcome depends on the structure of the starting allenic compound as well as the type of electrophile used [41,42,43,44]. Several diethylphosphono-substituted α-allenic alcohols [45] and glycols [46] were prepared by Brel [47,48] directly from alcohols by Horner-Mark rearrangement of unstable propargylic phosphites and used as starting materials for study of the cyclization in the presence of AgNO_3_ [46,47,49] and CuCl_2_ [48]. 

Our long-standing research program focuses on the development of efficient cyclization reactions of 1,1- [50,51] and 1,3-bifunctionalized allenes [52,53]. More specifically, our attention is drawn to phosphorylated hydroxyallenes as 1,1-bifunctionalized allenes that comprise a phosphoryl and a hydroxyalkyl group. The applications of these groups as temporary transformers of chemical reactivity of the allenic system in the synthesis of eventually heterocyclic compounds are of particular interest. These molecules can be considered a combination of an allenephosphonate or allenyl phosphine oxide and a hydroxyallene and they are supposed to have different reactivity profiles in cycloisomerization reactions. Our recent research has led to a significant result, whereby we have developed a convenient and efficient method for the regioselective synthesis of phosphorylated α-hydroxyallenes using an atom economical [2,3]-sigmatropic rearrangement [54]. In this paper, we present recent results of ongoing studies dedicated towards the optimization of the experimental conditions and the catalyst efficiency in the coinage metal salts-catalyzed cycloisomerization of α-hydroxyalkyl-allenephosphonates and phosphine oxides to 3-phosphorylated 2,5-dihydrofurans, which strongly improve the scope of this method. 

## 2. Results and Discussion

**Scheme 1:**
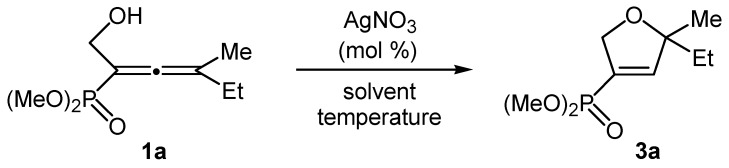
AgNO_3_-catalyzed cycloisomerization of the model dimethyl 1-hydroxymethyl-3-methylpenta-1,2-dienephosphonate (**1a**).

In addition to our previously reported preparation [55] of 2,5-dihydro-1,2-oxaphospholes by electrophilic cyclization of the 1-hydroxyalkyl-allenephosphonates **1** and allenyl phosphine oxides **2** due to the participation of the phosphonate neighboring group in the 5-*endo-trig* cyclization, we carried out the cycloisomerization reaction of the abovementioned compounds **1** and **2** in the presence of coinage metal salts as catalysts. Our initial work began with the cycloisomerization reaction of the model α-hydroxyalkyl-allenephosponate **1a** with AgNO_3_ in order to optimize the reaction conditions such as the influence of the solvent, the reaction temperature, the mol % and the type of the catalyst. The reaction occurred with formation of the dimethyl (5-ethyl-5-methyl-2,5-dihydrofuran-3-yl) phosphonate (**3a**, Scheme 1). The results are summarized in Table 1. 

**Table 1:** Optimization of the AgNO_3_-catalyzed cycloisomerization of the model dimethyl 1-hydroxymethyl-3-methylpenta-1,2-dienephosphonate (**1a**).

Entry	Solvent ^a^	Reaction temperature (°C)	AgNO_3_ (mol %)	Yield ^b^ (%)
1	ClCH_2_CH_2_Cl	−20	10	41
2	ClCH_2_CH_2_Cl	0	5	55
3	ClCH_2_CH_2_Cl	rt	5	63
4	ClCH_2_CH_2_Cl	reflux	5	45
5	CHCl_3_	rt	5	58
6	EtOH	rt	5	46
7	MeCN	rt	5	45
8	THF	rt	5	40
9	toluene	rt	5	28
10	acetone	rt	5	51
11	acetone/H_2_O	rt	5	75
12	acetone/H_2_O	rt	10	77
13	CH_2_Cl_2_	−20	5	72
14	CH_2_Cl_2_	rt	5	84 ^c^
15	CH_2_Cl_2_	rt	10	82

^a^ Reaction was carried out in the appropriate solvent (10 mL); ^b^ Yields determined by ^1^H and ^31^P-NMR analysis; ^c^ Isolated yield by chromatographic purification on silica gel.

**Scheme 2:**
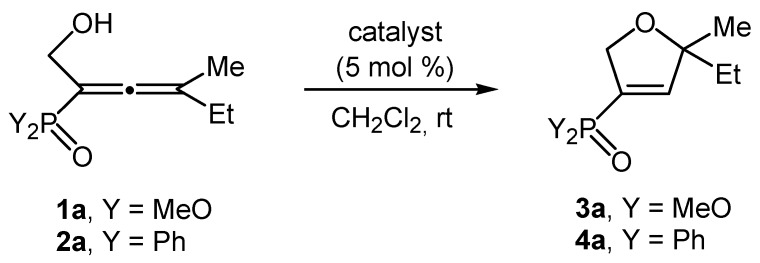
Coinage metal-catalyzed cycloisomerization of the model dimethyl 1-hydroxymethyl-3-methylpenta-1,2-dienephosphonate **1a** and the model 2-diphenylphosphinoyl-4-methylhexa-2,3-dien-1-ol **2a**.

At the very beginning, the reaction occurred in 1,2-dichloroethane at −20 °C with 10 mol % of catalyst (Table 1, entry 1). The yield was 41%. On the other hand, when we used 5 mol % of catalyst to carry out the reaction at 0 °C or room temperature in the same solvent the yield increased considerably (Table 1, entries 2 and 3). Lower yield was obtained at reflux in the same solvent (Table 1, entry 4). It is obvious that the optimal temperature for the cycloisomerization reaction of compound **1a** is room temperature. The use of polar solvents such as chloroform, ethanol, acetonitrile and THF at room temperature with 5 mol % AgNO_3_ produces the product in relatively good yields (Table 1, entries 5–8). A lower yield occurred in toluene (Table 1, entry 9). When the solvent was a mixture of acetone and H_2_O [15] at room temperature the yield was 77% with 5 mol % of catalyst (Table 1, entries 11 and 12). The data confirm that the optimal conditions for cycloisomerization of model compound **1a** (Table 1, entry 14) are methylene chloride, 5 mol % catalyst and room temperature. We found that reactions occurring at low or high reaction temperatures different from the optimum afford lower yields (Table 1, entries 2, 4 and 13). We also saw a lower yield when we used 10 mol % of catalyst at room temperature in dichloromethane (Table 1, entry 15). The type of catalyst and its influence on the yields of the cycloisomerization products of the α-hydroxyalkyl-allenephosphonates **1a**–**e** was also of great interest to us. We thus conducted a series of experiments to optimize the reaction conditions of the model compound **1a** (Scheme 2). We applied the following coinage metal salts as catalysts: AgNO_3_, AgClO_4_, AuCl, AuCl_3_, ZnCl_2_, NiCl_2_, PtCl_2_, SnCl_2_, AlCl_3_, PdCl_2_, Pd(PPh_3_)_4_, CuCl_2_, CuCl, CuBr, and CuI. The data reveal that both Au and Ag are excellent catalysts. Pd, Cu(II) and Pt are very good catalysts for our experiments. It becomes obvious that Zn, Ni, Sn and Al are relatively good catalysts. It is the Cu(I) catalysts that are bad. Table 2 presents the characteristics of all the above-mentioned catalysts in the cycloisomerization reaction of the model compound **1a**. 

The next step in our study was to explore the possibilities of the cycloisomerization reactions of the α-hydroxyalkyl-allenyl phosphine oxides **2a**–**e** synthesized by us. At the very beginning, we used 2-diphenylphosphinoyl-4-methylhexa-2,3-dien-1-ol (**2a**) as a model compound. The optimal cycloisomerization conditions included dichloromethane as solvent, 5 mol % catalyst and room temperature. The reaction occurred via an 5-*endo-trig* cyclization to give 4-(diphenylphosphinoyl)-2-ethyl-2-methyl-2,5-dihydrofuran (**4a**, Scheme 2). We performed a series of experiments with the sole intention of determining the best catalysts bearing in mind two criteria—highest yield and shortest reaction time. The model compound **2a** reacts with the catalysts shown in Table 2, in which the yields obtained and the reaction time of the preparation of isomer **4a** are presented. The experimental data reveals that the best catalytic characteristics correspond to Au, Ag and Pd catalysts (Table 2). The Cu(II), Zn and Pt catalysts that show very good results. The Sn, Al and Ni are relatively good. 

**Table 2:** Optimization of the coinage metal-catalyzed cycloisomerization of the model compound **1a** and the model dien-1-ol **2a**.

Entry	Catalyst	Reaction time ^a^ (min)		Yield ^b^ (%)	
		1a	2a	1a	2a
1	AuCl	20	30	97	91
2	AuCl_3_	30	35	94	89
3	AgClO_4_	30	55	83 ^c^	85 ^c^
4	AgNO_3_	50	65	80	80
5	PdCl_2_	100	115	73	80
6	Pd(PPh_3_)_4_	105	120	74	77
7	CuCl_2_	115	110	77	74
8	PtCl_2_	135	180	66	78
9	ZnCl_2_	160	135	50	75
10	NiCl_2_	225	395	53	36
11	SnCl_2_	310	255	38	57
12	AlCl_3_	345	340	34	32
13	CuCl	530	635	27	27
14	CuBr	545	690	29	22
15	CuI	600	725	24	23

^a^ On the average; ^b^ Yields determined by ^1^H- and ^31^P-NMR analysis; ^c^ Isolated yield by chromatographic purification on silica gel.

**Scheme 3:**
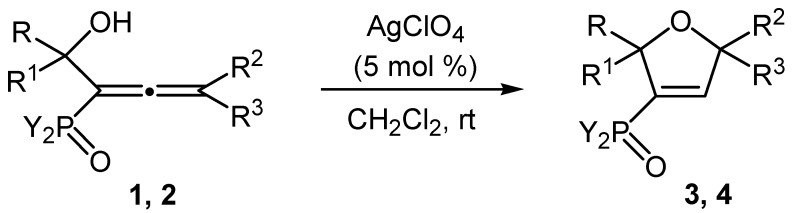
AgClO_4_-Catalyzed cycloisomerization of the phosphorylated α-hydroxyallenes **1** and **2**.

The investigation on the cycloisomerization reaction of the synthesized series of α-hydroxyalkyl-allenephosphonates **1a**–**e** was intended to be systematical. We applied the optimized reaction temperature, type of the solvent and molar ratio of the catalyst conditions in regard to the substrate. Having in mind that gold catalysts are expensive and sensitive to moisture, we decided to use AgClO_4_ as a main catalyst in cycloisomerization reaction in order to extend our study (Scheme 3). The results are explicit enough—a catalytic 5-*endo-trig* cycloisomerization occurs and the hydroxy group participates as an internal nucleophile to give the 2,5-dihydrofuran-3-yl phosphonates **3** in very good yields.

Our study was systematized also on the cycloisomerization of a series of α-hydroxyalkyl-allenyl phosphine oxides **2a**–**e** in methylene chloride at room temperature (Scheme 3). Moreover, it was the AgClO_4_ catalyst, which is easily accessible and relatively good, that was used in our research. The results are undisputable—the 3-diphenylphosphinoyl-2,5-dihydrofurans **4** are produced as a result of the participation of the neighboring hydroxy group as an internal nucleophile in the cyclization process. Table 3 shows the reaction times and yields of the cycloisomerization reaction of the α-hydroxyalkyl-allenephosphonates **1a**–**e** and allenyl phosphine oxides **2a**–**e**. 

**Table 3:** AgClO_4_-Catalyzed cycloisomerization of the phosphorylated α-hydroxyallenes **1** and **2**.

Entry	Allene	Y	R	R^1^	R^2^	R^3^	Reaction time ^a^ (min)	Product, Yield ^b^ (%)
1	**1a**	MeO	H	H	Me	Et	30	**3a**, 83
2	**1b**	MeO	H	H	Me	Bu	35	**3b**, 75
3	**1c**	MeO	H	H	-(CH_2_)_5_-		41	**3c**, 73
4	**1d**	MeO	H	Me	Me	Et	33	**3d**, 77
5	**1e**	MeO	Me	Me	Me	Bu	40	**3e**, 74
6	**2a**	Ph	H	H	Me	Et	55	**4a**, 85
7	**2b**	Ph	H	H	Me	Bu	58	**4b**, 85
8	**2c**	Ph	H	H	-(CH_2_)_5_-		75	**4c**, 82
9	**2d**	Ph	H	Me	Me	Et	57	**4d**, 84
10	**2e**	Ph	Me	Me	Me	Bu	64	**4e**, 82

^a^ On the average; ^b^ Isolated yield by chromatographic purification on silica gel.

## 3. Experimental Section 

### 3.1. General Information

All new synthesized compounds were purified by column chromatography and characterized on the basis of NMR, IR, and microanalytical data. NMR spectra were recorded on DRX Bruker Avance-250 (Bruker BioSpinGmbH, Karlsruhe, Germany) (^1^H at 250.1 MHz, ^13^C at 62.9 MHz, ^31^P at 101.2 MHz) and Bruker Avance II+600 (Bruker BioSpinGmbH) (^1^H at 600.1 MHz, ^13^C at 150.9 MHz, ^31^P at 242.9 MHz) spectrometers for solutions in CDCl_3_. All ^1^H- and ^13^C-NMR experiments were measured referring to the signal of internal TMS and ^31^P-NMR experiments were measured referring to the signal of external 85% H_3_PO_4_. *J* values are given in Hertz. IR spectra were recorded with an Afinity-1 FT-IR spectrophotometer (Shimadzu, Tokyo, Japan). Elemental analyses were carried out by the Microanalytical Service Laboratory of Faculty of Chemistry and Pharmacy, University of Sofia, Sofia, Bulgaria, using Vario EL3 CHNS(O) (Elementar Analysensysteme, Hanau, Germany). Column chromatography was performed on Kieselgel F_254_60 (70–230 mesh ASTM, 0.063–0.200 nm, Merck, Darmstadt, Germany). Reactions were carried out in oven dried glassware under an argon atmosphere and exclusion of moisture. All compounds were checked for purity on Kieselgel F_254_60 TLC plates (Merck).

### 3.2. Starting Materials

The starting phosphorylated α-hydroxyallenes **1** and **2** were prepared according to the established procedure [54]. CH_2_Cl_2_ was distilled over CaH_2_ and other organic solvents used in this study were dried over appropriate drying agents by standard methods and distilled prior to use. All other chemicals used in this study were commercially available and were used without additional purification unless otherwise noted. 

### 3.3. General Procedure for the Coinage Metal-catalyzed Cycloisomerization of the 1-Hydroxyalkyl-1,2-dienephosphonates ***1***

Metal salt catalyst (0.15 mmol) was added to a solution of the 1-hydroxyalkyl-1,2-dienephosphonate **1** (3.0 mmol) in dry dichloromethane (10 mL). The mixture was stirred at room temperature and in the dark for the minutes indicated in the Table 3. Saturated sodium chloride solution was added to precipitate the silver ions. The product was extracted by chloroform. The organic layer was dried over anhydrous sodium sulfate. After evaporation of the solvent, the residue was chromatographed on a column (silica gel, Kieselgel Merck 60 F_254_) with a mixture of ethyl acetate and hexane (6:1) as an eluent to give the pure products **3** as oils, which had the following properties:

*Dimethyl (5-ethyl-5-methyl-2,5-dihydrofuran-3-yl)phosphonate* (**3a**). This compound was obtained as a yellow oil, yield 83%. R_f_ 0.44; IR (neat, ν_max_, cm^−1^): 1128 (C-O-C), 1254 (P=O), 1627 (C=C). ^1^H-NMR (250.1 MHz): δ_H_ 0.89 (t, *J* = 7.4 Hz, 3H, Me-CH_2_), 1.32 (s, 3H, Me-C), 1.60-1.72 (m, 2H, Me-CH_2_), 3.76 (d, *J* = 12.2 Hz, 3H, MeO), 4.73–4.77 (m, 2H, CH_2_O), 6.53–6.57 (m, 1H, =CH). ^13^C-NMR (62.9 MHz) δ_C_ 8.6, 25.2 (*J* = 2.0 Hz), 33.0 (*J* = 4.5 Hz), 52.5 (*J* = 5.6 Hz), 74.9 (*J =* 20.5 Hz), 92.3 (*J =* 19.1 Hz), 127.5 (*J =* 195.9 Hz), 149.6 (*J =* 10.1 Hz). ^31^P-NMR (101.2 MHz): δ_P_ 15.9. Anal. Calcd for C_9_H_17_O_4_P requires: C 49.09, H 7.78. Found: C 49.12, H 7.74.

*Dimethyl (5-butyl-5-methyl-2,5-dihydrofuran-3-yl)phosphonate* (**3b**). This compound was obtained as a yellow oil, yield 75%. R_f_ 0.40; IR (neat, ν_max_, cm^−1^): 1120 (C-O-C), 1258 (P=O), 1626 (C=C). ^1^H-NMR (600.1 MHz): δ_H_ 0.88 (t, *J =* 7.2 Hz, 3H, Me-CH_2_), 1.19–1.26, 1.28–1.37, 1.56–1.67 (overlapping multiplets, 6H, (CH_2_)_3_-Me), 1.31 (s, 3H, Me-C), 3.75 (d, *J =* 12.0 Hz, 3H, MeO), 4.71–4.78 (m, 2H, CH_2_O), 6.54–6.57 (m, 1H, =CH). ^13^C-NMR (150.9 MHz) δ_C_14.1, 23.1, 25.5 (*J =* 2.1 Hz), 26.7, 40.0 (*J =* 4.5 Hz), 52.6 (*J =* 5.7 Hz), 74.8 (*J =* 20.6 Hz), 92.1 (*J =* 19.2 Hz), 127.6 (*J =* 196.1 Hz), 149.8 (*J =* 10.1 Hz). ^31^P-NMR (242.9 MHz): δ_P_ 16.0. Anal. Calcd for C_11_H_21_O_4_P requires: C 53.22, H 8.53. Found: C 53.18, H 8.55.

*Dimethyl (1-oxaspiro[4.5]dec-3-en-3-yl)phosphonate* (**3c**). This compound was obtained as an orange oil, yield 73%. R_f_ 0.60; IR (neat, ν_max_, cm^−1^): 1126 (C-O-C), 1257 (P=O), 1625 (C=C). ^1^H-NMR (250.1 MHz): δ_H_ 1.22–1.45, 1.4–1.72, 1.8–2.02 (overlapping multiplets, 10H, (CH_2_)_5_), 3.77 (d, *J =* 12.5 Hz, 3H, MeO), 4.73–4.75 (m, 2H, CH_2_O), 6.72–6.75 (m, 1H, =CH). ^13^C-NMR (62.9 MHz) δ_C_23.4, 25.8, 35.4 (*J =* 4.5 Hz), 52.6 (*J =* 5.6 Hz), 73.8 (*J =* 20.8 Hz), 91.4 (*J =* 19.1 Hz), 126.4.5 (*J =* 196.2 Hz), 149.9 (*J =* 9.8 Hz). ^31^P-NMR (101.2 MHz): δ_P_ 17.1. Anal. Calcd for C_11_H_19_O_4_P requires: C 53.65, H 7.78. Found: C 53.62, H 7.70.

*Dimethyl (2-methyl-1-oxaspiro[4.5]dec-3-en-3-yl)phosphonate* (**3d**). This compound was obtained as an orange oil, yield 77%. R_f_ 0.59; IR (neat, ν_max_, cm^−1^): 1119 (C-O-C), 1251 (P=O), 1624 (C=C). ^1^H-NMR (600.1 MHz): δ_H_ 1.21–1.31, 1.34–1.52, 1.68–1.89 (overlapping multiplets, 10H, (CH_2_)_5_), 1.38 (d, *J =* 6.4 Hz, 3H, Me-CH), 3.75 (d, *J =* 11.2 Hz, 3H, MeO), 5.01–5.05 (m, 1H, Me-CH), 6.75–6.79 (m, 1H, =CH). ^13^C-NMR (150.9 MHz) δ_C_ 20.5 (*J =* 9.9 Hz), 20.6 (*J =* 4.5 Hz), 23.1, 26.8, 34.9 (*J =* 4.6 Hz), 52.5 (*J =* 5.7 Hz), 74.2 (*J =* 20.5 Hz), 91.9 (*J =* 19.3 Hz), 127.0 (*J =* 196.0 Hz), 149.7 (*J =* 10.0 Hz). ^31^P-NMR (242.9 MHz): δ_P_ 17.0. Anal. Calcd for C_12_H_21_O_4_P requires: C 55.38, H 8.13. Found: C 55.44, H 8.09.

*Dimethyl (5-butyl-2,2,5-trimethyl-2,5-dihydrofuran-3-yl)phosphonate* (**3e**). This compound was obtained as a yellow oil, yield 74%. R_f_ 0.56; IR (neat, ν_max_, cm^−1^): 1117 (C-O-C), 1257 (P=O), 1622 (C=C). ^1^H-NMR (600.1 MHz): δ_H_ 0.88 (t, *J =* 7.1 Hz, 3H, Me-CH_2_), 1.19–1.39, 1.41–1.53, 1.56–1.65 (overlapping multiplets, 6H, Me-(CH_2_)_3_), 1.33 (s, 3H, Me-C), 1.44, 1.46 (ss, 6H, Me_2_C), 3.76 (d, *J =* 11.2 Hz, 3H, MeO), 6.57–6.59 (m, 1H, =CH). ^13^C-NMR (150.9 MHz) δ_C_14.2, 23.5, 25.7 (*J =* 2.0 Hz), 27.1, 28.5, 28.7, 41.1 (*J =* 4.6 Hz), 52.5 (*J =* 5.8 Hz), 74.9 (*J =* 20.4 Hz), 89.7 (*J =* 19.2 Hz), 127.1 (*J =* 196.1 Hz), 150.3 (*J =* 10.1 Hz). ^31^P-NMR (242.9 MHz): δ_P_ 17.2. Anal. Calcd for C_13_H_25_O_4_P requires: C 56.51, H 9.12. Found: C 56.57, H 9.17.

### 3.4. General Procedure for the Coinage Metal-catalyzed Cycloisomerization of the 2-Diphenylphosphinoyl-2,3-dien-1-ols ***2a-c*** and the 3-Diphenylphosphinoyl-3,4-dien-2-ols ***2d,e***

Metal salt catalyst (0.15 mmol) was added to a solution of the 2-diphenylphosphinoyl-2,3-dien-1-ols **2a**–**c** or the 3-diphenylphosphinoyl-3,4-dien-2-ols **2d**,**e** (3.0 mmol) in dry dichloromethane (10 mL). The mixture was stirred at room temperature and in the dark for the minutes indicated in the Table 3. Saturated sodium chloride solution was added to precipitate the metal ions. The product was extracted by dichloromethane. The organic layer was dried over anhydrous sodium sulfate. The solvent was removed using a rotatory evaporator and the residue was purified by column chromatography (silica gel, Kieselgel Merck 60 F_254_) with a mixture of ethyl acetate and hexane (5:1). The pure products **4** had the following properties:

*Diphenyl (5-ethyl-5-methyl-2,5-dihydrofuran-3-yl) phosphine oxide* (**4a**). This compound was obtained as a colourless oil, yield 85%. R_f_ 0.58; IR (neat, ν_max_, cm^−1^): 1121 (C-O-C), 1174 (P=O), 1436, 1483 (Ph), 1620 (C=C). ^1^H-NMR (600.1 MHz): δ_H_ 0.91 (t, *J =* 7.5 Hz, 3H, Me-CH_2_), 1.33 (s, 3H, Me-C), 1.61–1.73 (m, 2H, Me-CH_2_), 4.78–4.86 (m, 2H, CH_2_O), 6.27–6.31 (m, 1H, =CH), 7.39–7.78 (m, 10H, 2Ph). ^13^C-NMR (150.9 MHz) δ_C_ 8.98, 25.3 (*J =* 2.1 Hz), 33.0 (*J =* 4.4 Hz), 75.5 (*J =* 17.1 Hz), 92.7 (*J =* 14.9 Hz), 133.5 (*J =* 104.0 Hz), 128.1–132.9, 149.6 (*J =* 7.3 Hz). ^31^P-NMR (242.9 MHz): δ_P_ 22.2. Anal. Calcd for C_19_H_21_O_2_P requires: C 73.06, H 6.78. Found: C 73.02, H 6.83.

*Diphenyl (5-butyl-5-methyl-2,5-dihydrofuran-3-yl) phosphine oxide* (**4b**). This compound was obtained as a colourless oil, yield 85%. R_f_ 0.52; IR (neat, ν_max_, cm^−1^): 1120 (C-O-C), 1178 (P=O), 1437, 1492 (Ph), 1618 (C=C). ^1^H-NMR (600.1 MHz): δ_H_ 0.89 (t, *J =* 7.1 Hz, 3H, Me-CH_2_), 1.22–1.41, 1.59–1.69 (overlapping multiplets, 6H, (CH_2_)_3_-Me), 1.34 (s, 3H, Me-C), 4.77–4.84 (m, 2H, CH_2_O), 6.28–6.31 (m, 1H, =CH), 7.47–7.72 (m, 10H, 2Ph). ^13^C-NMR (150.9 MHz) δ_C_ 14.1, 23.8, 25.0, 25.8 (*J =* 2.0 Hz), 40.2 (*J =* 4.6 Hz), 75.3 (*J =* 17.0 Hz), 92.4 (*J =* 15.0 Hz), 128.7–132.4, 133.7 (*J =* 103.6 Hz), 149.7 (*J =* 7.3 Hz). ^31^P-NMR (242.9 MHz): δ_P_ 21.0. Anal. Calcd for C_21_H_25_O_2_P requires: C 74.10, H 7.40. Found: C 74.05, H 7.46.

*Diphenyl (1-oxaspiro[4.5]dec-3-en-3-yl) phosphine oxide* (**4c**). This compound was obtained as a yellow oil, yield 82%. R_f_ 0.55; IR (neat, ν_max_, cm^−1^): 1120 (C-O-C), 1169 (P=O), 1439, 1491 (Ph), 1622 (C=C). ^1^H-NMR (600.1 MHz): δ_H_ 1.32–1.38, 1.47–1.55, 1.64–1.74 (overlapping multiplets, 10H, (CH_2_)_5_), 4.77–4.79 (m, 2H, CH_2_O), 6.45–6.50 (m, 1H, =CH), 7.48–7.70 (m, 10H, 2Ph). ^13^C-NMR (150.9 MHz) δ_C_ 23.0, 25.3, 35.8 (*J =* 4.4 Hz), 74.3 (*J =* 17.0 Hz), 91.6 (*J =* 14.8 Hz), 128.3–134.2, 133.0 (*J =* 103.7 Hz), 149.8 (*J =* 7.3 Hz). ^31^P-NMR (242.9 MHz): δ_P_ 21.2. Anal. Calcd for C_21_H_23_O_2_P requires: C 74.54, H 6.85. Found: C 74.50, H 6.79.

*Diphenyl (2-methyl-1-oxaspiro[4.5]dec-3-en-3-yl) phosphine oxide* (**4d**). This compound was obtained as a yellow oil, yield 84%. R_f_ 0.47; IR (neat, ν_max_, cm^−1^): 1119 (C-O-C), 1173 (P=O), 1439, 1485 (Ph), 1621 (C=C). ^1^H-NMR (600.1 MHz): δ_H_ 1.18 (d, *J =* 6.4 Hz, 3H, Me-CH), 1.32–1.38, 1.42–1.54, 1.55–1.64 (overlapping multiplets, 10H, (CH_2_)_5_), 5.06–5.09 (m, 1H, Me-CH), 6.97–6.96 (m, 1H, =CH), 7.30–7.69 (m, 10H, 2Ph). ^13^C-NMR (150.9 MHz) δ_C_ 20.2 (*J =* 9.8 Hz), 20.5 (*J =* 4.6 Hz), 22.2, 26.5, 34.7 (*J =* 4.7 Hz), 82.4 (*J =* 15.8 Hz), 89.8 (*J =* 15.3 Hz), 128.4–135.0, 136.8 (*J =* 102.4 Hz), 149.7 (*J =* 7.8 Hz). ^31^P-NMR (242.9 MHz): δ_P_ 22.2. Anal. Calcd for C_22_H_25_O_2_P requires: C 74.98, H 7.15. Found: C 75.05, H 7.11.

*Diphenyl (5-butyl-2,2,5-trimethyl-2,5-dihydrofuran-3-yl) phosphine oxide* **(4e)**. This compound was obtained as a colourless oil, yield 82%. R_f_ 0.46; IR (neat, ν_max_, cm^−1^): 1115 (C-O-C), 1163 (P=O), 1438, 1483 (Ph), 1624 (C=C). ^1^H NMR (600.1 MHz): δ_H_ 0.87 (t, *J =* 7.2 Hz, 3H, Me-CH_2_), 1.27–1.36, 1.49–1.54 (overlapping multiplets, 6H, (CH_2_)_3_-Me), 1.32 (s, 3H, Me-C), 1.45, 1.48 (ss, 6H, Me_2_C), 6.90–6.96 (m, 1H, =CH), 7.46–7.79 (m, 10H, 2Ph). ^13^C-NMR (150.9 MHz) δ_C_ 14.1, 23.8, 25.0, 26.3 (*J =* 2.1 Hz), 27.5, 27.7, 40.3 (*J =* 4.7 Hz), 83.5 (*J =* 16.0 Hz), 91.0 (*J =* 14.5 Hz), 128.6–134.7, 133.5 (*J =* 103.4 Hz), 144.9 (*J =* 7.6 Hz). ^31^P-NMR (242.9 MHz): δ_P_ 22.1. Anal. Calcd for C_23_H_29_O_2_P requires: C 74.98, H 7.93. Found: C 74.93, H 8.01.

## 4. Conclusions 

In conclusion, we have developed a coinage metal-catalyzed cycloisomerization of phosphorylated α-hydroxyallenes, which provides an efficient route to 3-phosphorylated 2,5-dihydrofurans. Due to the ready availability of the starting materials and the catalyst, the convenient operation and mild conditions (room temperature, short reaction time), the very good yields and the usefulness of the resulting 2,5-dihydrofuran products, the reaction shows potential and will be useful in organic synthesis as well in the application of the method in target-oriented synthesis. Further investigation on the chemistry of other phosphorylated allenols for the synthesis of different heterocyclic systems is being intensively carried out in our laboratory. Moreover, results of an initial investigation of the biological activity of the compounds prepared were encouraging, and the antibacterial and antifungal activities of selected compounds are now under investigation in our University.
